# Combined Use of Systematic Conservation Planning, Species Distribution Modelling, and Connectivity Analysis Reveals Severe Conservation Gaps in a Megadiverse Country (Peru)

**DOI:** 10.1371/journal.pone.0114367

**Published:** 2014-12-05

**Authors:** Javier Fajardo, Janeth Lessmann, Elisa Bonaccorso, Christian Devenish, Jesús Muñoz

**Affiliations:** 1 Centro Universitario de Mérida, Universidad de Extremadura, Mérida, Spain; 2 Real Jardín Botánico (RJB-CSIC), Madrid, Spain; 3 Centro de Biodiversidad y Cambio Climático, Universidad Tecnológica Indoamérica, Quito, Ecuador; 4 Biodiversity Institute, University of Kansas, Lawrence, Kansas, United States of America; 5 Division of Biology and Conservation Ecology, Manchester Metropolitan University, Manchester, United Kingdom; 6 Centro de Ornitología y Biodiversidad (CORBIDI), Lima, Peru; U.S. Geological Survey, United States of America

## Abstract

Conservation planning is crucial for megadiverse countries where biodiversity is coupled with incomplete reserve systems and limited resources to invest in conservation. Using Peru as an example of a megadiverse country, we asked whether the national system of protected areas satisfies biodiversity conservation needs. Further, to complement the existing reserve system, we identified and prioritized potential conservation areas using a combination of species distribution modeling, conservation planning and connectivity analysis. Based on a set of 2,869 species, including mammals, birds, amphibians, reptiles, butterflies, and plants, we used species distribution models to represent species' geographic ranges to reduce the effect of biased sampling and partial knowledge about species' distributions. A site-selection algorithm then searched for efficient and complementary proposals, based on the above distributions, for a more representative system of protection. Finally, we incorporated connectivity among areas in an innovative post-hoc analysis to prioritize those areas maximizing connectivity within the system. Our results highlight severe conservation gaps in the Coastal and Andean regions, and we propose several areas, which are not currently covered by the existing network of protected areas. Our approach helps to find areas that contribute to creating a more representative, connected and efficient network.

## Introduction

Protected areas represent the cornerstone of conservation strategies to protect biodiversity *in situ* [1]. Although several approaches to conservation planning exist, there is consensus regarding the importance of focusing on representativeness [2], which means that reserves need to account for the full variety of biodiversity, and provide conditions to safeguard it against the processes that threaten its persistence [3]. A wealth of discussion has arisen in the last few decades on whether current protected areas fulfill conservation goals with global proposals promoting an increase in the amount of protected land worldwide [4]. As neither threats nor biodiversity are equally distributed on Earth, conservation planning should consider parameters such as biodiversity richness, endemism, and threat, in addition to the size of protected area systems [5]. These considerations are relevant because classical reserve selection criteria, usually based on opportunity, aesthetics or politics, cannot always guarantee biodiversity conservation [3], [6].

Systematic approaches to conservation have arisen in recent years to assist the creation of new protected areas by proposing objective criteria for deciding where, why, and how conservation efforts and resources need to be directed in order to obtain maximized benefits and more representative protection networks [3], [7], [8]. Systematic conservation planning is a multistep procedure that includes (1) the statement of clearly defined conservation goals, (2) the evaluation of current protected area systems in achieving such goals and the detection of conservation gaps, and (3) the proposition of priority areas for conservation, whose protection will contribute to meeting the declared goals and addressing identified deficiencies [9]. In this context, site selection and decision-support algorithms propose areas that maximize the achievement of conservation goals, whilst minimizing resources expended, subject to the constraint that all features (species or systems) meet their conservation goals [3], [10]. Systematic planning is particularly timely in biodiversity rich tropical regions that are challenged by high deforestation rates and usually have incipient reserve systems, which are not subjected to network design analyses. Such systems are of unknown efficiency, further constraining system development [11]–[14].

To justify where to place protected areas, reserve selection algorithms require information on species distributions and threats across the territory. Species distributions are often incorporated through direct use of census data in the form of point occurrences [14]. This data is stored in natural history collections, which are increasingly easier to access through public databases. However, except for well studied regions, available data is usually sparse, incomplete and spatially biased, generally incorporating many omission errors that lead to the underestimation of distributions [14], [15]. Thus, available information for biodiversity patterns of tropical countries generally lacks the detail and quality required to be used by conservation planners. To deal with the above problems associated with species data, species distributions models (SDMs) are becoming widely used in conservation biology as an approximation to species ranges. These predictions are made by relating known occurrences to a set of meaningful environmental predictors. Although SDMs are not free of error and uncertainty [16], [17], if due care is taken, they can help reduce the impact of sampling bias and data sparseness [14]. Therefore, the integration of species distribution modeling and systematic conservation planning has shown great potential to select representative and efficient conservation areas [11], [18]–[20].

In conservation planning, setting priorities within the portfolio of proposed conservation areas is an important, often overlooked step. Not all the potential areas have the same characteristics, protect the same species or have the same urgency for protection [9]. Additionally, priority analyses become especially relevant when working at the country or regional scale, since it is not feasible to implement reserves from all potential areas. A qualitative ranking assessment for the proposed areas may guide the decision of what to protect first. Prioritization might be based on several criteria and, among them, we consider that connectivity is a crucial one that is often ignored. Having a connected network of protected areas (i.e., a system where the location among constituent units allows the movement of organisms across them) is important for the conservation of species, in particular those with large territorial requirements, whose protection might not be afforded by singular protected areas [1]. However, site-selection algorithms may only consider connectivity at a basic level, providing control over the compactness of the proposals by minimizing the area/perimeter ratio of resulting areas [21]. To enhance this control, we propose the incorporation of an innovative measure of connectivity [22] as a post-hoc analysis to prioritize the protection of those areas that increase the connectivity of the network.

Peru is one of 17 megadiverse countries [23] and includes the Tropical Andes, and Tumbes-Chocó-Magdalena biodiversity hotspots [24]. The National System of State Protected Areas (SINANPE) provides protection to 195,288 km^2^ (15.2%) of Peru's territory, and aims to protect a representative sample of the country's biodiversity [25]. However, since large conservation gaps have been identified, the system needs to be revised and extended [26], [27]. In the years 2000 and 2009, priority areas for conservation were proposed at national scale, mostly based on expert criteria and focusing on conservation of high species diversity and endemism [27], [28], particular groups of organisms (e.gr., birds) [29] or marine biodiversity [30], [31].

To date, site selection algorithms have not been used for identifying terrestrial priority areas for conservation at a national scale in Peru. Some conservation studies have used eco-regions as conservation targets at a regional scale [32], [33]. Unfortunately, these studies are too narrow in scope, and do not provide guidelines at national scale, which restricts their significance on integrated decision-making processes. There are also global scale studies that may provide a general framework for the identification of global priority areas in Peru [34], but their scale and scope are too broad, which makes them inappropriate for the country or regional scale. Hence, Peru is lacking an integrated study focused on species representation using decision support software for identifying conservation priorities. This situation is not specific to Peru, but common in developing countries harboring most of the biodiversity hotspots and most of the relatively undisturbed areas suitable for biodiversity conservation.

We believe that conservation approaches based on representativeness should not be disregarded by state policies, and that selection algorithms combined with connectivity analysis may provide recommendations to increase protection systems in an efficient and complementary manner. Thus, the aim of this paper is to evaluate the degree to which the existing national protected area network fulfills the biodiversity needs of the country, and to identify areas of maximized suitability for conservation to complement the existing network, using Peru as an example of megadiverse country. We approach these challenges by using species of several groups of terrestrial organisms as biodiversity indicators, complementing previous studies. Species distribution models were used as surrogate inputs of species ranges, and connectivity was explicitly considered to prioritize the proposed conservation areas.

## Materials and Methods

### Study area

**Figure 1:**
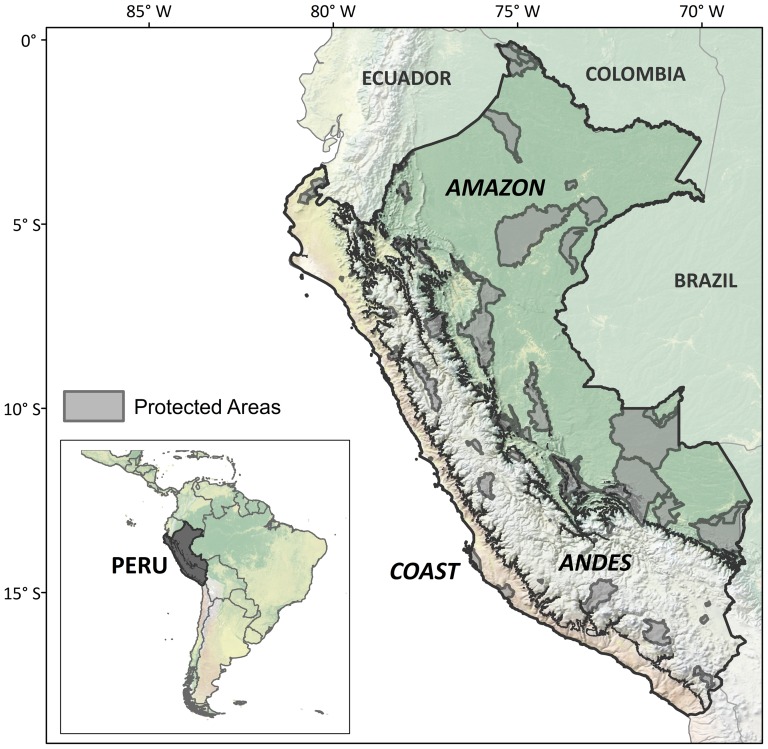
Study area. Peru's geographical regions and protected areas included in the study.

Continental Peru covers 1,277,206 km^2^, and can be divided into three main geographical regions: the Pacific coast to the west (‘Coast’), the mountains of the Andean cordillera, running the length of the country from south to north (‘Andes’), and the Amazonian rainforests to the east (‘Amazon’) (Figure 1). Peru's National System of State Protected Areas (SINANPE) covers 15.2% of the territory in almost 100 reserves [25]. For this study, after excluding marine reserves, we considered a total of 77 protected areas as the system to be analyzed, covering 14.3% of the country. This set includes the 72 continental national protected areas and 5 regional protected areas.

### Conservation features

#### Species data

Ideally, a representative approach aims to protect biodiversity as a whole; however, in practice it is impossible to include all species in an analysis. Thus, biodiversity surrogates need to be used. We used a set of species as biodiversity surrogates of the elements to be conserved (i.e., conservation features) within the protected area network. In order to achieve maximum representation of biodiversity, we tried to include the largest possible number of species from several taxonomic groups, threat level, and geographic extent. A total of 2,869 terrestrial species were included, corresponding to 133 amphibians, 74 reptiles, 185 mammals, 1,163 birds, 1,226 vascular plants (i.e. Arecaceae, Bignoniaceae, Bromeliaceae, Fabaceae, Lauraceae, and Rubiaceae), and 88 Helicoiine butterfly taxa (Table 1, Appendix S1). Birds were the best represented group in the study, and reptiles had the highest percentage of threatened species. Species' occurrence data was collated in a database from museums, online data sets, researchers' personal data sets and literature. More details about the conservation features are available in the Appendix S2. Occurrence data from online databases is known to include a certain amount of error and bias [35]. Although the large number of conservation features used here prevented us from an exhaustive analysis of the data, we reviewed the database for taxonomy errors and eliminated problems detected with the georeferencing of occurrence data.

**Table 1:** Summary of species data.

	Species in this study^1^	% of the Peru total in the study^2^	% of the threatened^3^	Species on the Coast^4^	Species on the Andes^4^	Species on the Amazon^4^
**AMPHIBIANS**	133	25	17	7	31	98
**BIRDS**	1163	64	44	205	554	595
**BUTTERFLIES**	88	-	-	9	29	52
**MAMMALS**	185	36	49	24	48	136
**REPTILES**	74	21	50	19	25	34
**PLANTS**	1226	7	-	167	505	698
Bignoniaceae	64	-	-	9	12	52
Bromeliaceae	143	-	-	26	105	45
Fabaceae	345	-	-	98	104	192
Gesneriaceae	94	-	-	3	50	44
Lauraceae	129	-	-	9	55	74
Arecaceae	76	-	-	0	0	76
Rubiaceae	375	-	-	22	179	215
**TOTAL**	**2869**			**431**	**1192**	**1613**

1 Total number of species included in the dataset.

2 Percentage of the total number of species present for that group in Peru.

3 Percentage of threatened species in the dataset.

4 Number of species from each region. Note that species may be present in more than one region. For this reason, the sum of species in all three regions is larger than the total number of species in the study.

#### Species' Distributions

We generated species distribution models from species' occurrence data using Maxent [36], a machine-learning algorithm based on the principle of maximum entropy [37], [38]. Maxent performs well modelling presence-only occurrence data with low sample sizes, and with moderate errors in their georeferencing, making it especially suitable for our species data [39]–[41]. Fifteen of the Worldclim 1.4 bioclimatic variables (http://www.worldclim.org) were used as predictor variables at a 1 km^2^ spatial resolution [42], representing current climatic variables of potential biological relevance [43] (Appendix S2). The remaining four variables were excluded from the modeling process because they show ovoid shaped, unrealistic patterns over eastern Peru, which result from the inherent limitations of the interpolation algorithms where only few meteorological stations were available [44]. As we were interested in obtaining the best possible models and not in explaining what variables are important for each species, we did not attempt to remove correlated predictors. Instead, we took advantage of Maxent's capacity for choosing the most informative variables among all predictors for modeling each species [45], [46]. Besides, Maxent is known to make robust predictions even if there is collinearity among variables [46], [47].

We used Maxent 3.3.3k with the following settings: convergence threshold set to 0.00001; number of background points to 10,000; maximum iterations to 500; and regularization parameter to ‘auto’, to allow the program to select an amount of regularization that is appropriate for climate and locality data [37]. Based on studies of Maxent performance with low sample sizes [41], [48]–[50], species with fewer than 10 occurrence records (minimum of five) were only modelled in cases where published information on the species' ranges enabled us to verify the resulting distribution maps. Maxent models were reclassified to presence/absence predictions using the “Maximum Training Sensitivity Plus Specificity” threshold, which has proven to generally produce more accurate results than other thresholds [51], [52]. As a further control measure, we discarded 257 species' models that differed largely from distributional ranges reported in the literature [53]–[55] or that had an AUC lower than 0.85 (calculated using 10-fold cross-validation). Also, 25 models were edited slightly to eliminate small areas unlikely to be occupied by the species due to geographic barriers. We stacked the 2,869 individual distribution models to obtain richness maps by taxon group that were qualitatively compared with richness patterns of Peru available in the literature [56], [57].

### Conservation goals

In site-selection terminology, a species conservation goal is the amount of a species' range that must be included within a reserve system in order for it to be considered as sufficiently protected. In the present study, goals were calculated separately for each species, to acknowledge differences in their life history, current conservation status, and perceived importance in conservation measures. The goal for each conservation feature was calculated as the sum of two partial goals:

*Distribution size goal*: We assigned a more demanding representation goal to species with more restricted ranges, acknowledging the negative relationship between species distribution size and extinction risk [12]. The value given to each species was scaled between a minimum coverage of 5% for species with a geographic distribution equal to or greater than 200,000 km^2^ in Peru, and a maximum of 25% for species with ranges equal to or less than 1,000 km^2^ as in Rodrigues et al. [34]. The 200,000 km^2^ upper threshold corresponds to the range size observed in one third of the species in our data set.
*Conservation status goal*: We assigned goals to species identified as threatened by the IUCN [55] following a decreasing scale: Critically Endangered (CR), 25%; Endangered (EN), 17.5%; Vulnerable (VU), 10%; Near Threatened (NT), 5%; Least Concern (LC), Not Evaluated (NE), and Data Deficient (DD), 0%. We recognize that NE and DD species might be of conservation concern, but having no further information on their status, we decided not to increase their goals arbitrarily.

The final goals ranged between 5% for the species with lesser conservation needs (large distributions and Least Concern classification) to almost 50% for Critically Endangered species with small distributions (see Appendix S1).

### Gap analysis

We performed a species-focused gap analysis [9] to evaluate how the current Peruvian protected area network accomplishes the proposed conservation goals. For each species, we calculated the percentage of its SDM occurring inside protected areas and compared it with its conservation goal (Appendix S1). For this comparison, we organized the species by taxonomic group, threat status, and geographic region. Species are considered insufficiently protected by the current protected areas system when percent coverage is below their conservation goal.

### Identification of priority areas for conservation

We used Marxan 2.4.3 [58] to identify the most efficient set of areas that, if protected, would make the network of protected areas more representative of the species under study and, by extension, of Peruvian biodiversity. Marxan uses the *minimum set approach* to identify a portfolio of priority conservation areas, minimizing the area needed to accomplish previously established conservation goals [59] with the least investment of resources (see Appendix S2). We used 97,499 square planning units (PUs) of 16 km^2^. Each PU is associated with data on species occurrence within it, base cost, and edge length. Base cost was estimated using the Human Footprint index [60] in recognition that PUs with less human influence are cheaper to conserve. The boundary length modifier (BLM) was optimized to 300, which offers an efficient tradeoff between reserve boundary length and the size of priority areas, following Stewart and Possingham [61]. Also, PUs coinciding with current protected areas were forced to be selected in the solutions. Marxan analysis was conducted using the simulated annealing algorithm followed by an iterative improvement and 100 replicates. We delimited proposed priority conservation areas from Marxan's summed solution. The summed solution represents the number of times each PU was included in all 100 replicate solutions, describing the utility of a PU in building efficient representative solutions [21]. Priority areas were delimited from PUs which were selected 75 or more times [21], or between 50 and 74 times, when they were spatially contiguous to one or more blocks of PUs selected over 75 times.

### Prioritization within proposed conservation areas

In recognition that protecting all the proposed areas in the short term is unrealistic, we prioritized the resulting areas according to three important criteria for decision making. This approach allows us to provide recommendations on where conservation efforts need to be directed first. Thus, areas resulting optimal for conservation were ranked according to three factors: (1) *selection frequency in additional scenarios*, (2) *vulnerability*, and (3) *connectivity*. Each factor was scored for all proposed areas based on the mean value of all PUs within it.

The *selection frequency in additional scenarios score* represents the importance of a proposed area under different conservation scenarios. To calculate this score, we ran Marxan with 10 additional scenarios where all parameters were left as described in the previous section with the exception of the conservation goals, which were multiplied by the following 10 factors: 0.2, 0.4, 0.6, 0.8, 1.0, 1.2, 1.4, 1.6, 1.8, 2.0. Each run of Marxan produced 100 solutions and a summed solution made up of the selection frequency across the 100 runs. Finally, we summed the 10 Marxan summed solutions to produce the index, which ranges from 0 to 1000. This score represents the averaged frequency of selection across all scenarios. As a result, areas with high scores are formed by PUs that were selected across several scenarios and with varying conservation goals.
The *vulnerability score* highlights impacted areas with higher urgency for protection. To calculate the score we used the Human Footprint Index as a measure of the human influence on each PU. Where PUs coincided with mining areas, the score was increased to a high value equaling that of cities.
The *connectivity score* favors proposed conservation areas that increase connectivity among the conservation area system. It is based on the probability of connectivity index (dPC) [62] which quantifies the amount of available habitat in the landscape for a particular species, accounting both for the habitat inside an area itself (intra-patch connectivity) and between areas (inter-patch connectivity) [22], [62], [63]. We calculated dPC using Conefor Sensinode 2.6 (available at http://www.conefor.org/) [64], considering a network among both existing protected areas and proposed conservation areas. We calculated distances between elements of the system as ‘effective distances' using Pathmatrix 1.1 [65]. ‘Effective distances’ are a measure of distance modified by the cost of moving across a resistance surface [66], which is able to assess more realistically the movement of medium to large dispersers between areas. The human footprint layer [60] and the presence of mining were used as a resistance surface for distance calculations under the premise that movement across less disturbed areas is easier than across impacted ones and impossible across mines.

The three scores were normalized to values between 0 and 100, and summed to give each proposed area an overall priority score. More details about the calculation of each score are provided in Appendix S2. Areas were classified as high, medium, and low priority using natural breaks in the priority score [67].

## Results

### Species richness patterns

The most species rich regions resulting from the 2,869 species distribution models are the Amazonian humid forests of Loreto and Madre de Dios departments, along with the Andes-Amazon transition in the central and northern Andean cordillera. The Coastal region, with a relatively low number of species throughout, and the Altiplano in the southern Andes were the poorest regions. This pattern is common to plants, birds, and butterflies, with mammals, amphibians, and reptiles clearly richer in the forests of the Amazonian lowlands. However, the coastal area is more relevant for reptiles, especially in the north.

### Achievement of conservation goals in the current protected area system

We found that 843 species, 29% of the total, are insufficiently protected in the current reserve system with relation to the defined conservation goals, while 71% of the taxa studied are well represented (Table 2). Reptiles, butterflies, and plants are the groups less satisfactorily protected with 53%, 43%, and 36%, of their species under protected, respectively. Mammals and birds meet conservation goals the best, with 20% and 22% of species insufficiently protected, respectively.

**Table 2:** Species representation in the current protected area network of continental Peru based on the conservation goals defined in this study. Results are classified by taxonomic group, IUCN category and region.

Category		species protected (conservation goals met)	species under protected (conservation goals not met)
**Group**	**Plants**	93 (70%)	40 (30%)
	**Amphibians**	909 (78%)	254 (22%)
	**Reptiles**	51 (58%)	37 (42%)
	**Birds**	148 (80%)	37 (20%)
	**Mammals**	37 (50%)	37 (50%)
	**Butterflies**	788 (64%)	438 (36%)
**UICN**	**CR**	0 (0%)	10 (100%)
	**EN**	4 (14%)	24 (86%)
	**VU**	26 (38%)	42 (61%)
	**NT**	37 (53%)	33 (47%)
	**LC**	1106 (83%)	233 (17%)
	**DD**	16 (67%)	8 (33%)
	**NE**	837 (63%)	493 (37%)
**Region**	**Coast**	174 (40%)	257 (60%)
	**Andes**	757 (64%)	435 (36%)
	**Amazon**	1387 (86%)	226 (14%)
**Total**		**2026 (71%)**	**843 (29%)**

CR: Critically Endangered, EN: Endangered, VU: Vulnerable, NT: Near Threatened, LC: Least Concern, DD: Data Deficient, NE: Not evaluated.

We found that threatened species are not as well protected as non-threatened: all the Critically Endangered, 86% of the Endangered, and 62% of the Vulnerable species did not achieve their conservation goals. Non-threatened categories (Near Threatened and Least Concern), have an adequate coverage for at least 50% of their species. Analysis by geographical region shows that the least protected species occur on the Coast, followed by the Andes, with 60% and 36% of species showing insufficient coverage, respectively, while most of the Amazonian species (86%) are adequately protected. However, in terms of numbers of species, the Andes (435) have more underprotected species than the Coast (257) or the Amazon (226).

### Identification of priority areas for conservation

**Figure 2:**
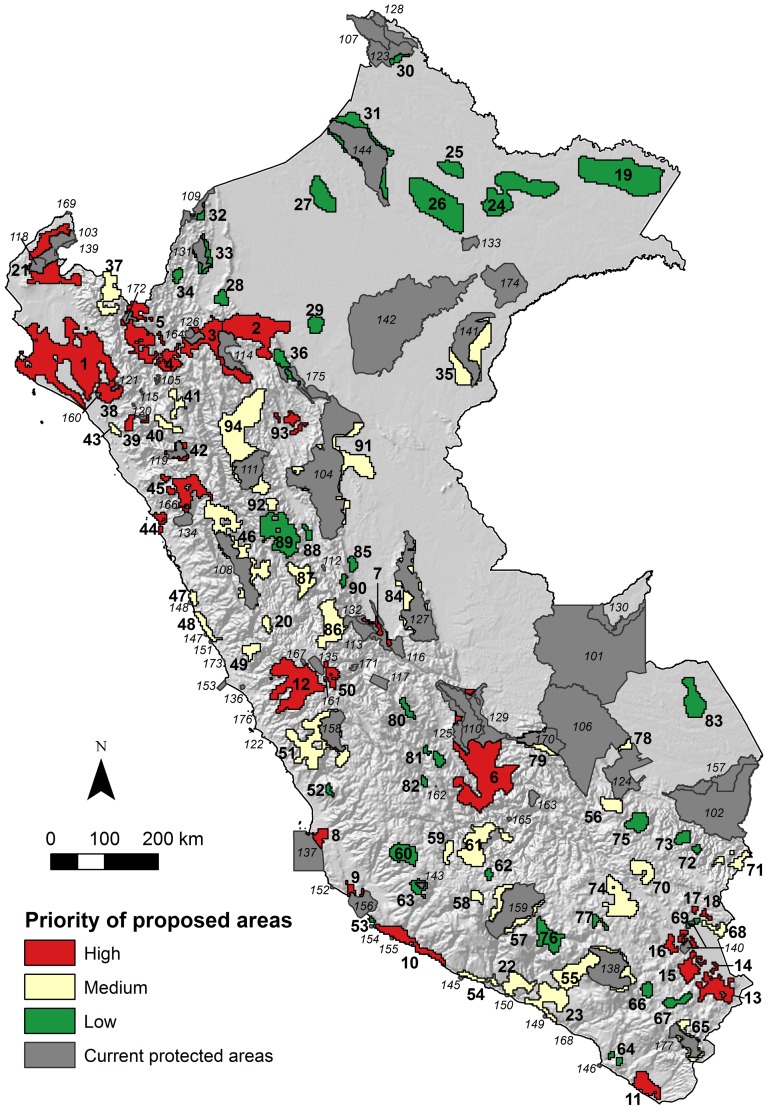
Current protected areas in Peru and proposed areas for conservation illustrating global prioritization. The final rank is a combination of the three priority criteria: selection frequency of PUs across scenarios, vulnerability and connectivity. See Table 3 for key to proposed areas and protected areas.

Based on Marxan's summed solution, we identified 94 areas of maximum suitability for conservation across the country (Table 3, Figure 2). Together, those areas represent almost 160,000 km^2^, 12% of continental Peru, representing almost the same percentage of the country already under protection. Of these 94 areas, 66 (70%) are independent from existing protected areas, 28 (30%) are extensions of existing protected areas, and nine (10%) could act as corridors between existing reserves. Fifty-three areas are less than 1,000 km^2^ in size, while eight are larger than 5,000 km^2^.

**Table 3:** List of the proposed conservation areas (with an indicative name based on the department or protected area they are part of) and the existing protected areas used in the analysis.

**PROPOSED CONSERVATION AREAS**	58. New PA Ayacucho 1	121. Laquipampa WR
1. New PA Piura-Lambayeque coast	59. New PA Ayacucho 2	122. Los Pantanos de Villa WR
2. Extension PF Alto Mayo	60. New PA Ayacucho 3	123. Airo Pai CR
3. Connector PF Alto Mayo – NS Cordillera de Colán	61. New PA Apurímac-Ayachucho	124. Amarakaeri CR
4. New PA North Andes	62. New PA Apurímac	125. Ashaninka CR
5. Extension Tabaconas-Namballe NS	63. Extension Pampa Galeras Barbara D'Achille NR	126. ChayuNaín CR
6. Extension Otishi NP	64. New PA Moquegua 1	127. El Sira CR
7. Extension San Matías-San Carlos PF	65. Extension Vilacota Maure RPA	128. Huimeki CR
8. Extension Paracas NR	66. New PA Moquegua 2	129. Machiguenga CR
9. Extension San Fernando NR	67. New PA Moquegua-Puno	130. Purus CR
10. New PA Arequipa coast 1	68. New PA Titicaca 6	131. Tuntanain CR
11. New PA Tacna	69. Extension North Titicaca NR	132. Yanesha CR
12. New PA Lima-Pasco-Junín	70. New PA Puno 1	133. Allpahuayo Mishana NR
13. New PA Titicaca 1	71. New PA Puno 2	134. Calipuy NR
14. New PA Titicaca 2	72. New PA Puno 3	135. Junín NR
15. New PA Titicaca 3	73. New PA Puno 4	136. Lachay NR
16. Extension South Titicaca NR	74. New PA Cusco-Puno 1	137. Paracas NR
17. New PA Titicaca 4	75. New PA Cusco-Puno 2	138. Salinas and Aguada Blanca NR
18. New PA Titicaca 5	76. New PA Arequipa Andes 1	139. Tumbes NR
19. New PA Loreto 1	77. New PA Arequipa Andes 2	140. Titicaca NR
20. New PA Ancash-Lima	78. Connector Amarakaeri CR-Manu NP	141. Matsés NR
21. Extension Cerros de Amotape NP	79. Extension Megantoni NS	142. Pacaya Samiria NR
22. New PA Arequipa coast 3	80. New PA Junín 2	143. Pampa Galeras Barbara D' Achille NR
23. New PA Arequipa coast 4	81. New PA Ayacucho 4	144. Pucacuro NR
24. New PA Loreto 2	82. New PA Huancavelica	145. Punta Atico NR
25. New PA Loreto 3	83. New PA Madre de Dios	146. Punta Coles NR
26. New PA Loreto 4	84. Extension El Sira CR	147. Punta Colorado NR
27. New PA Loreto 5	85. New PA Ucayali	148. Punta Culebras NR
28. New PA Loreto 6	86. New PA Huánuco-Pasco	149. Punta Hornillos NR
29. New PA Loreto 7	87. New PA Huánuco 1	150. Punta La Chira NR
30. Extension Airo Pai CR	88. New PA Huánuco 2	151. Punta La Litera NR
31. Extension NR Pucacuro	89. New PA Huánuco 3	152. Punta Lomitas NR
32. Extension NP Ichigkat Muja – Cordillera del Cóndor	90. New PA Huánuco 4	153. Punta Salinas, Isla Huampanú and Isla Mazorca NR
33. Extension RC Tuntanain	91. Extension Cordillera Azul NP	154. Punta San Juan NR
34. New PA Amazonas	92. New PA San Martín 1	155. Punta Lomas NR
35. Extension RV Matsés	93. New PA San Martín 2	156. San Fernando NR
36. Extension RPA Cordillera Escalera	94. Extension Río Abiseo NP	157. Tambopata NR
37. New PA Piura	**EXISTING PROTECTED AREAS**	158. Nor Yauyos-Cochas LR
38. Connector Bosque de Pómac HS-Laquipamba WR	101. Alto Purus NP	159. Subcuenca del Cotahuasi LR
39. Extension Bosques nublados de Udima WR	102. Bahuaja Sonene NP	160. Bosque de Pómac HS
40. New PA South Cajamarca 1	103. Cerros de Amotape NP	161. Chacamarca HS
41. New PA South Cajamarca 2	104. Cordillera Azul NP	162. Pampa de Ayacucho HS
42. Extension Sunchubamba HP	105. Cutervo NP	163. Machupicchu HS
43. New PA Lambayeque-La Libertad	106. Manu NP	164. Cordillera de Colán NS
44. New PA La Libertad coast	107. Güeppi-Sekime NP	165. Ampay NS
45. New PA La Libertad Andes	108. Huascarán NP	166. Calipuy NS
46. Extension Huascarán NP	109. Ichigkat Muja-Cordillera del Cóndor NP	167. Huayllay NS
47. Extension Punta Culebras NR	110. Otishi NP	168. Lagunas de Mejía NS
48. New PA Ancash South coast	111. Río Abiseo NP	169. Los Manglares de Tumbes NS
49. New PA Lima	112. Tingo María NP	170. Megantoni NS
50. New PA Junín	113. Yanachaga-Chemillén NP	171. Pampa Hermosa NS
51. Extension Nor Yauyos-Cochas LR	114. Alto Mayo PF	172. Tabaconas-Namballe NS
52. New PA Ica North	115. Pagaibamba PF	173. Albúfera de Medio Mundo RPA
53. New PA Ica South	116. San Matias-San Carlos PF	174. Comunal Tamshiyacu Tahuayo RPA
54. New PA Arequipa coast 2	117. Pui Pui PF	175. Cordillera Escalera RPA
55. Extension Salinas and Aguada Blanca NR	118. El Angolo HR	176. Humedales de Ventanilla RPA
56. New PA Cusco	119. Sunchubamba HR	
57. Extension Cotahuasi Subcuenca LR	120. Bosques Nublados de Udima WR	

PA: Proposed Conservation Area; NP: National Park; NR: National Reserve; CR: Communal Reserve; LS: Landscape Reserve; PF: Protection Forest; WR: Wildlife Refuge; NS: National Sanctuary; HS: Historical Sanctuary, HP: Hunting Preserve; RPA: Regional Protected Area.

### Prioritization of the proposed conservation areas

**Figure 3:**
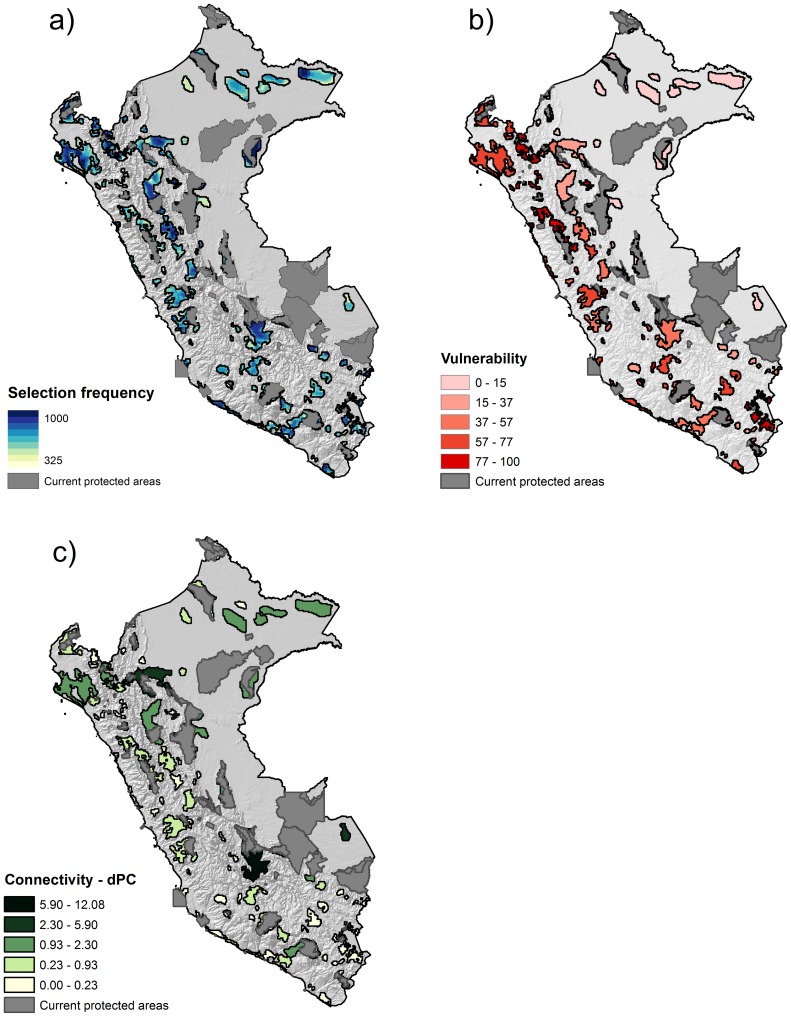
Priority assessment. Maps showing the three criteria used to evaluate the conservation priority of the proposed conservation areas in this study: a) selection frequency of the planning units, including additional solutions with varying conservation goals; b) vulnerability, derived from the Human Footprint index and mining concessions; c) dPC connectivity index.

The prioritization criteria ranked the proposed areas according to their importance in different conservation scenarios, their vulnerability, their importance to connect the protected area system (Figure 3), and their overall priority (Figure 2). There are 26 proposed areas of high priority, 33 of medium priority, and 35 of low priority. High priority areas are more abundant in the Coastal and Andean regions, especially towards the north, while most of Amazonian areas remain of low priority.

Increasing and decreasing species conservation goals, as implemented in the additional Marxan scenarios, showed that varying the goals does not have a large influence on the location of the proposed conservation areas. Not surprisingly, the higher the goals, the larger the area included in the solution. The summation of the 10 scenarios produced nested structures, with centers formed by highly selected PUs across scenarios surrounded by PUs selected only in a few scenarios (Figure 3a).

The vulnerability of the proposed conservation areas, as measured by the vulnerability score, increases gradually from east to west (Figure 3b). Proposed areas in the Amazon region have the lowest *vulnerability score* because they occur in less impacted areas of Peru. To the contrary, proposed areas in the Andes and on the Coast, where human influence is higher, are the most vulnerable.

Proposed conservation areas with a central position in the network and located next to other protected areas have higher *connectivity scores* (Figure 3c) because they represent connectors between protected areas and the proposed areas selected by Marxan. Also, larger areas had higher connectivity scores, because the dPC index is influenced by the amount of habitat available inside each patch.

## Discussion

Designing and complementing protection networks to safeguard biodiversity is a difficult task for governments and conservationists in megadiverse countries. As such, Peru is challenged by the combination of high biodiversity, information gaps, and limited resources in enlarging its incomplete network of protected areas [28], emphasizing the need for conservation planning.

The set of 2,869 species used in this study resulted in patterns of potential richness congruent with the regional patterns found by Bass et al. [56], a broader scale study including mammals, amphibians and plant species. Although knowledge of such patterns of biodiversity is still incipient and not devoid of uncertainty, such similarity in overall results suggests that they are consistent with currently described Peruvian biodiversity patterns.

### Adequacy of current protected area system in Peru

We found that the national system of protected areas does not provide sufficient protection for a large number of species according to the specified conservation goals. Thus, our conclusions emphasize the need for creating new reserves to complement the existing ones.

According to our results, the three geographical regions of the country have important conservation gaps. However, we observed differences in the degree of protection among them. In terms of proportions, species on the coast are the worst represented in the current protected area system, whereas Amazonian species are the best represented, and Andean species are between the two. This pattern is probably the result of two factors. First, the Coastal and Andean regions have a much higher human population density (they include the five largest cities of the country), which severely impacts biodiversity and limits conservation opportunities. Second, conservation actions have been traditionally biased towards the Amazonian ecosystems because they have higher species richness, rainforests are prioritized internationally, and funding is more readily obtainable to protect them [26], [28]. The six largest protected areas in Peru, each of them more than 10,000 km^2^ in extension, are in the Amazon or in the Andes-Amazon transition area, while reserves on the Coast are fewer and smaller, with an average size of 180 km^2^. In fact, 18 protected areas on the coast are smaller than 50 km^2^. This pattern contrasts strongly with the fact that the Coastal region has always been considered important for conservation by Peruvian scientists, and was included in early conservation plans, which were later overridden when external funds focused on Amazonian conservation [26]. This pattern of protection results in mammals and birds being the most protected groups, both with centers of species richness in the Amazon, while reptiles, with many species in the Coastal region, represent the least protected group. To the contrary, in terms of species numbers, the Coast and the Amazon have similar conservation gaps (257 and 226 insufficiently protected species, respectively), and thus both regions could be considered to be of similar conservation importance. Nevertheless, when compared to the Amazon, the number of under-protected species on the Coast represents a much higher percentage of its diversity, and the loss of these species would be more critical to the conservation of the coastal region's diversity.

Our results also revealed that threatened species (CR, EN and VU) are the least protected, with higher endangerment categories translating to lower achievement of the species' conservation needs. Threatened species included in our study are likely to have high conservation goals because both components making up the goal are likely to be high, that is, a threatened status and a reduced range size. These high conservation goals are justified from a conservationist point of view, but are difficult to attain because they need large proportions of species' ranges to be protected.

In congruence with our findings, previous studies based on eco-regions found that 60% of them do not meet the 10% protection goal proposed at the IVth World Congress on National Parks and Protected Areas [68]. Other results, consistent with the present study, indicate that the protected area network is biased towards Amazonian ecosystems and humid forests, and call for a more balanced protected area system [27].

### Proposed areas for conservation

We identified 94 priority areas for conservation that may be used in decision support processes to expand Peru's national protected area network (Figure 2). They are complementary to the national systems of protected areas [25] and protecting any of them would contribute to creating a more representative system because they fill conservation gaps by increasing the coverage of under-protected species. Most of the newly proposed areas are in the Andean and Coastal regions, and a few of them are in the Amazon. Increasing protection of the two former regions would balance, in some measure, the current bias towards the Amazon. Given the infeasibility of including all the proposed areas in the national protected area system, implying an extension of 186% of the system or that a quarter of Peru's terrestrial area would be under protection, we ranked the proposed areas following systematic criteria. The resulting priorities may guide decision makers on where to focus efforts on extending the current system (Figures 2 and 3).

Our study shows that, in general, high priority areas are concentrated in the Coast and in the Andes, mainly because they are the most transformed regions of the country. Although not many conservation opportunities are left in these regions, this combination of unique biodiversity and threat requires urgent protection measures. Some proposed areas in these regions have relatively high human impact, but are still compatible with conservation if efficient protection strategies are implemented. To the north, our results point to a high priority area covering the coasts of Piura and Lambayeque (Figure 2, area n° 1) including, among other species and ecosystems, the San Pedro de Vice mangrove forest. This is one of the few mangrove remnants in Peru and represents a link with those in southern Ecuador, which has previously been highlighted [27]. Additionally, extensions of the Paracas and San Fernando National Reserves (Figure 2, areas n° 8 and 9), two areas on the coasts of Arequipa and Tacna (Figure 2, areas n° 10 and 11), a large area east of Lima (Figure 2 area n° 12), or the set of small areas around Titicaca Lake (Figure 2, areas n° 13, 14, 15, 16, 17 and 18), are also high priority elements. Further, some of the newly proposed areas are of high to medium priority because they contribute highly to connectivity among existing areas, or newly proposed ones. For example, the areas 2, 3, 4, and 5 (Figure 2) form a corridor in the northern Andes, from the Loreto lowlands to the Ecuadorian border. All these areas represent opportunities for clustering larger protection elements, thus providing more effective protection for organisms with large home ranges as well as ecosystem functions [69]. On the other hand, the newly proposed areas in the Amazon are of low priority because they are isolated and have lower vulnerability. Nevertheless, almost all the Peruvian Amazon is under concession for oil exploitation, what might increase the vulnerability of these forests. Protected areas are in practice the only portions of the Amazon which remain outside oil concessions [70].

Five of the proposed priority areas (Figure 2, areas n° 1, 3, 13, 19, and 20) coincide with formal ‘Reserved Zones’ or potential protected areas waiting to be categorized and declared and where resource exploitation has been interrupted. Our results support the inclusion of these five areas in the national protected area system. Additionally, this study found that some of the 55 privately-owned protected areas (covering a marginal 0.17% of the territory) are of high or medium priority. In addition to highlighting the importance of these private reserves, our results may guide individuals and conservation organizations in the establishment of new reserves, maximizing the impact of funding.

Many of the proposed areas (Figure 2, areas n° 1, 2, 4, 5, 12, 13, 19, 21, 22, and 23) were also considered important by previous studies, regardless as to whether species [28], [29] or under-represented ecosystems and species [27] were used as conservation features. Such concurrence confirms that the proposed areas here have a sound grounding and should direct future conservation endeavors in Peru. Nevertheless, our study also differs from previous findings as a consequence of using a different approach. While Rodriguez and Young [28] and SERNANP [27] focus on species richness and thus stressed conservation of the Amazon formations, we searched for a protected area system that explicitly emphasizes representativeness, complementarity, and connectivity at a country scale. We did not find large conservation gaps in the Amazon and thus propose efforts be oriented to improving protection in the Coastal and Andean regions given the limited funding in Peru, a situation which is common in other developing countries.

With regards the methods, we believe that the procedure proposed here for incorporating connectivity into conservation planning represents a valuable contribution. The relevance of connectivity as a key element for conservation has been highlighted before [71] but, to date, selection algorithms (including Marxan) only include this concept at a basic level. The control provided by Marxan's BLM parameter is only a partial solution to incorporating connectivity, given that it provides for limited adjustment of the spatial compactness of the areas proposed for conservation, what is sometimes referred to as the structural connectivity of the areas. Yet, it is not capable of taking into consideration the spatial relationship, including isolation, between parts of the network [21]. The analysis presented here was able to rank selected areas for conservation according to their importance for connectivity, providing valuable information for the creation of more connected networks. However, connectivity was implemented as a *post hoc* analysis and had no influence on the selection of conservation areas. Hence, we encourage further research to incorporate connectivity within the selection algorithm for priority areas.

Another finding related to the methods employed also merits discussion due to its conservation implications. When we produced the 10 additional Marxan solutions by increasing or decreasing the conservation goals, we found that the total area of each solution was directly related to how large the goals were. Interestingly, all the solutions retained the same set of core areas, but were proportional in size to the size of the conservation goals, agreeing with previous studies [72]. The similarity in core areas is beneficial because it provides flexibility in the expansion of the protected area system. If funding is a limitation, policy-makers may start by establishing reserves in the core areas, with the confidence that they are always selected whether conservation goals are high or low, and that subsequently they may act as seeding areas for future enlargement of the system.

### Final Considerations

Systematic conservation planning in megadiverse countries is challenging. Their high diversity implies collecting information for a large number of species, while the available distribution data available is still scarce for many of them and gaps in knowledge are common. It is important to continue building up natural history collections and making them accessible online, as well as maintaining efforts to reduce the bias in global databases [35]. Even though it will remain virtually impossible to gather a satisfactory amount of information with acceptable quality for all species in the short term, the urgency to reduce biodiversity loss obliges the immediate use of currently available information by conservation planners. In this context, the use of SDM is vital to reduce the impact of sampling biases. However, working with such a large number of species is a difficult task, with corresponding sacrifices to methodological improvements (e.g., using species-specific backgrounds [73] or using ensemble modeling [74]) due to automation of analysis. Thus, given the uncertainty associated with the species data and the resulting SDMs, we recommend implementing field validations and rapid biological inventories in the priority areas as a preparatory step to their establishment as protected areas.

Finally, we found that the achievement of the conservation goals of such a large number of species inevitably involves protecting large expanses of land. Although Peru, Ecuador, and Venezuela are among the countries with the highest percentage of their territory protected (14%, 19% and 17%, respectively), their conservation gaps are still large at the species level, as was shown by Lessmann et al. [75], Delgado-Jaramillo [76], and confirmed here. Therefore, it is important to include a prioritization analysis of the proposed conservation areas to provide recommendations on where conservation efforts need to be directed first.

We are well aware that site-selection algorithms are intended to help users to make informed decisions, not to exclude them from the decision-making process. Planning tools are decision-support systems that offer recommendations and orientation about what to protect, but are not decision-making systems themselves [77]. The areas that we recommend for protection, as well as their prioritization, can be thought of as a preliminary portfolio that needs to be debated by authorities, conservationists, land-owners, settlers, and stakeholders. Additional information, including socioeconomic constraints, establishment and management costs, fine-filter threats or opportunities for restoration must be incorporated so that decisions can be taken in a consensual manner.

## Supporting Information

Appendix S1 List of species included in the analyses, assigned conservation goals and distribution information.
(XLSX)

Appendix S2 Detailed Methods.
(DOCX)
