# Correction: Combined Use of Systematic Conservation Planning, Species Distribution Modelling, and Connectivity Analysis Reveals Severe Conservation Gaps in a Megadiverse Country (Peru)

**DOI:** 10.1371/journal.pone.0122159

**Published:** 2015-03-06

**Authors:** The PLOS ONE Staff

There are errors in Table 2. The complete, correct Table 2 can be viewed here.

**Table 2:** Species representation in the current protected area network of continental Peru based on the conservation goals defined in this study. Results are classified by taxonomic group, IUCN category and region.

Category		species protected (conservation goals met)	species under protected (conservation goals not met)
**Group**	**Amphibians**	93 (70%)	40 (30%)
	**Birds**	909 (78%)	254 (22%)
	**Butterflies**	51 (58%)	37 (42%)
	**Mammals**	148 (80%)	37 (20%)
	**Reptiles**	37 (50%)	37 (50%)
	**Plants**	788 (64%)	438 (36%)
**UICN**	**CR**	0 (0%)	10 (100%)
	**EN**	4 (14%)	24 (86%)
	**VU**	26 (38%)	42 (62%)
	**NT**	37 (53%)	33 (47%)
	**LC**	1106 (83%)	233 (17%)
	**DD**	16 (67%)	8 (33%)
	**NE**	837 (63%)	493 (37%)
**Region**	**Coast**	174 (40%)	257 (60%)
	**Andes**	757 (64%)	435 (36%)
	**Amazon**	1387 (86%)	226 (14%)
**Total**		**2026 (71%)**	**843 (29%)**

CR: Critically Endangered, EN: Endangered, VU: Vulnerable, NT: Near Threatened, LC: Least Concern, DD: Data Deficient, NE: Not evaluated

There are errors in the second sentence in the Results subsection titled “Achievement of conservation goals in the current protected area system.” The correct sentence is: Reptiles, butterflies, and plants are the groups less satisfactorily protected with 50%, 42%, and 36%, of their species under protected, respectively.
