# Supplementary material for: Combined Use of Systematic Conservation Planning, Species Distribution Modelling, and Connectivity Analysis Reveals Severe Conservation Gaps in a Megadiverse Country (Peru)
Source: PLoS One. 2014 Dec 5;9(12):e114367. doi: 10.1371/journal.pone.0114367 (PMC4257666; doi:10.1371/journal.pone.0114367)
Supplement: Appendix S2 — Detailed Methods. (DOCX) [file pone.0114367.s002.docx]

**SUPPORTING INFORMATION**

**Appendix S2: Detailed Methods**

1. **Species data**

A total of 2,869 terrestrial species were included, corresponding to 132 amphibians, 74 reptiles, 185 mammals, 1,163 birds, 88 butterflies, and 1,227 vascular plants from Arecaceae, Bignoniaceae, Bromeliaceae, Fabaceae, Lauraceae, and Rubiaceae (Table S1). From the total of species of each group in Peru, the species included here represent 25% of amphibians (http://www.amphibiaweb.org/), 21% of reptiles [1], 64% of birds [2], 36% of mammals [3], 7% of vascular plants [1], while the proportion of the Heliconiine butterflies represent a small and indeterminable representation of Lepidoptera. Regarding threatened status, the dataset included 17% of all Peruvian threatened amphibians, 44% of threatened birds, 49% of threatened mammals and 50% of threatened reptiles [4], while the butterfly and plant groups categorization is still not complete in the global IUCN red list. Regarding the regional distributions of the species, 14% of them are from the Coast, 37% of the Andes and 50% of the Amazon, the three regions that can be recognized in Peru.

The occurrence records were obtained from the following museums, institutions and databases: Aarhus University Palm Transect Database (AU), Academy of Natural Sciences, Department of Herpetology (ANSP), American Museum of Natural History (AMNH), British Museum Natural History (BMNH), California Academy of Sciences (CAS), Centro de Datos para la conservación, Facultad de Ciencias Forestales de la Universidad Nacional Agraria La Molina (CDC-UNALM), Christian Devenish personal collection (CDPC), Cornell University (CU), Delaware Museum of Natural History (DMNH), eBIRD dataset (eBIRD), Field Museum (FMNH) and its Rapid Biological Inventories (RBI), Florida Museum of Natural History (FLMNH), Instituto de Biología, Universidad Nacional Autónoma de Mexico (IBUNAM), Instituto de Ciencias Naturales, Universidad Nacional de Colombia (ICN), Louisiana State University (LSUMZ), Missouri Botanical Garden Tropicos Database (MBG), Museo Civico di Storia Naturale di Milano (MSNM), Museo de Zoología de la Universidad Tecnológica Indoamérica (MZUTI), Museum d’Histoire Naturelle (MHNG), Museum of Comparative Zoology, Harvard University (MCZ), National Museum of Natural History from the Smithsonian Institution (USNM), National University Mayor de San Marcos Museum (NUMSM), Natural History Museum of Los Angeles County (LACM), Natural History Museum of Utah (UMNH), Royal Ontario Museum (ROM), Santa Barbara Museum of Natural History (SBMNH), Texas Cooperative Wildlife Collection (TCWC), Michigan State University (MSU), Universiteit van Amsterdam, Zoologisch Museum (ZMA), Universitets København, Zoologisk Museum (ZMUC), University of California (MVZ), University of Kansas, Museum of Natural History (KU), University of Michigan, Museum of Zoology (UMMZ), Western Foundation of Vertebrate Zoology (WFVZ), William E. Duellman field series (WED), Yale Peabody Museum of Natural History (YPM). Many of the databases were accessed through MANIS (http://manisnet.org/), ORNIS 2 (http://ornis2.ornisnet.org/), HERPNET2 (http://herpnet2.org/), GBIF (http://www.gbif.org/) or IABIN (http://www.oas.org/en/sedi/dsd/iabin/) data portals. Records from the publications Duellman and Lehr [5] and Rosser et al. [6] were also used.

For Heliconiine butterflies, subspecies rank was used instead of species because this taxonomic level is the commonly used for the group, there is a geographic component in their recognition and has conservation value in itself. The bird’s database included observational records from eBird database [7]. Use of this non-vouchered data is justified because birds are relatively easy to recognize in the field, and many birdwatchers can accurately identify them [8], resulting in a wealth of information that largely increased the representativeness of this group in the analysis. However, as this kind of data can potentially contain a larger proportion of errors, all occurrences were examined and compared with Schulenberg et al. [2], and records outside the accepted distribution were not used.

1. **Species distribution models**

We generated species distribution models (SDM) from species’ occurrence data [9,10] using Maxent 3.3.3k [11]. We used the following fifteen Worldclim 1.4 bioclimatic variables at 1 km^2^ resolution: annual mean temperature (bio1), mean diurnal range (bio2), isothermality (bio3), temperature seasonality (bio4), maximum temperature of the warmest month (bio5), minimum temperature of the coldest month (bio6), temperature annual range (bio7), mean temperature of the wettest quarter (bio10), mean temperature of the driest quarter (bio11), annual precipitation (bio12), precipitation of the wettest month (bio13), precipitation of the driest month (bio14), precipitation seasonality (bio15), precipitation of the wettest quarter (bio16) and precipitation of the driest quarter (bio17) [http://www.worldclim.org, 12]. The variables mean temperature of the wettest quarter (bio 08), mean temperature of driest quarter (bio 09), precipitation of the warmest quarter (bio 18) and precipitation of the coldest quarter (bio 19) were excluded from the modeling process because they show unrealistic patterns over eastern Peru

After control measures based on AUC and comparison with the literature, 257 species models were discarded and removed from the dataset used in subsequent steps. The referred value of 2,869 models already acknowledges the exclusion of discarded models, and therefore corresponds to the final number of species included in the analyses.

1. **Identification of priority areas for conservation: Marxan**

We used Marxan 2.4.3 [13] to identify areas where conservation suitability is maximized, meaning that they would make the network of protected areas more efficient and representative of existing biodiversity. Marxan uses the *minimum set approach*, defined as the minimal subset of units that efficiently accomplish previously established goals [14], accounting for proposals that are efficient with the least investment of resources. Marxan solves the minimum set problem by dividing the landscape into ‘planning units’ (PUs), small parcels which could potentially be included in (or excluded from) the final solution.

In order to find good solutions (i.e., collections of PUs), Marxan needs to compare the cost and goals achieved of many possible solutions with some basal score. This is accomplished by the use of a mathematical objective function that gives a score to each solution based on several costs and penalties [14]:

*Score* = ∑*_PUs_Cost* + *BLM* ∑*_PUs_Boundary* + ∑*_Goals_Penalty* Eq. 1

The most desirable solution has the lowest score, because it is formed by ‘cheaper’ sites, has a shorter boundary length and meets all the species’ conservation goals proposed. We parameterize each term of the objective function as follows:

*PU’s base cost.* Human influence was considered as an ecological cost for PUs, and was used to favor the selection of lower perturbed regions under the assumption that they are more appropriate for conservation. The human influence was obtained from the Human Footprint Index [15,16], that takes into account effective human population density, land transformation, accessibility, and electrical power infrastructure of the territory, in an index scaled from 0 to 100 where high values indicate negative effects over biodiversity. For this reason, PUs with a cost over 32, corresponding with highly impacted areas as cities, their surroundings, or heavily transited highways, where considered not appropriate for conservation and where excluded from possible solutions. Additionally, PUs coinciding with mining concessions were considered incompatible with conservation and their base cost was increased up to 32 (the same value that is found in cities) in order to remove them from the eligible choices.

*Edge length.* This parameter penalizes sets of PUs with large perimeter and high fragmentation, because those conditions have negative effects over diversity and makes conservation more difficult [14]. The boundary length modifier (BLM) is the parameter used to control for compactness of the reserve system and it was optimized for a correct level of spatial compactness to 300, following Steward and Possingham [17] recommendations.

*Penalty*. The penalty for not reaching conservation goals increases the cost of the solution as compared with situations in which conservation goals are met, forcing Marxan to search for combinations of PUs achieving goals with the lowest total score (Eq. 1). We assigned a Penalty Factor (SPF) value of 1 for each species, satisfactorily achieving the defined goals in preliminary testing runs.

Additionally, PUs coinciding with current protected areas were tagged in a way that forced the algorithm to select them in the solutions, because that allowed us to search for solutions that complement what the current network of protection already protects and to avoid redundancies.

Marxan was executed 100 times conducting 10,000,000 iterations per execution, in order to find and compare 100 solutions. Summing these 100 solutions we obtained Marxan’s “summed solution”, which was used to delimit conservation areas. In this last solution, each PU has a selection frequency that ranges between 0 and 100, according to the times it was selected in a Marxan solution across the 100 runs. Marxan was executed using the simulated annealing algorithm followed by an iterative improvement and the following parameters: 100 runs, 10,000,000 iterations per simulation, 10,000 temperature decreases per simulation, and an adaptive initial temperature and cooling factor.

1. **Prioritization within proposed conservation areas**

The priority areas obtained from Marxan runs were ranked according to: (1) *the frequency in additional scenarios score*, (2) the *vulnerability score*, and (3) the *connectivity* *score*.

*Frequency in additional scenarios score*: It represents the importance of a proposed area under different conservation scenarios. This score characterizes the averaged frequency of selection across 10 Marxan summed solutions. Each scenario was constructed by varying species conservation goals, while all other parameters remained constant, and equal to those described in the previous section of the general Marxan execution. For each scenario, the set of species conservation goals was multiplied by one of the following 10 factors: 0.2, 0.4, 0.6, 0.8, 1.0, 1.2, 1.4, 1.6, 1.8, 2.0 (to be noticed that the 1.0 scenario is equivalent to the execution that was used to delimit conservation areas, as conservation goals remained the same). Each scenario returned a summed solution (with selection frequencies ranging from 0 to 100), and the 10 summed solutions were summed up to build this index, which ranges from 0 to 1000. We calculated this score to overtake the certain degree of arbitrariness that species conservation goals have by exploring the result of modulated goals. As a result, areas with high scores are formed by PUs that were selected across several scenarios, meaning that it is versatile for conservation purposes. Protecting them is a good investment in every conservation situation.

*Vulnerability score*: The vulnerability of a proposed area is defined by the impact of human activities on it, and is the average of the human footprint index of the included PUs. When a PU is located on highly disturbed areas, it faces more threats and the biodiversity that it hosts is less likely to persist, making the urgency for its protection greater than in low impacted areas. Prioritizing areas of high threat is a reactive approach to vulnerability in opposition to proactive conservation, which gives more importance to wild areas where there are more conservation opportunities [18]. We preferred the reactive approach as a measure of urgency, although we recognize it may conflict with feasibility. Nevertheless, Marxan only includes in its solutions areas with moderate disturbance where conservation is still possible because places impacted beyond recovery are prevented of being chosen. Moderately disturbed areas might be of high importance for conservation if they host species that do not occur in other places, and where losing even tiny patches of remnant habitat may end in their extinction [18].

*Connectivity score*: For a system of reserves to be effective, patches of protected habitat must be connected among them. When the moment to decide where to place a new reserve comes, it is relevant to acknowledge whether a candidate area will improve connectivity among the system. The topology of the elements in the system and the characteristics of the intervening non-protected area through which potential functional connections are established, are factors conditioning connectivity [19]. We evaluated the connectivity importance of each priority area using the probability of connectivity index (dPC), which is based on the ‘habitat availability’ concept [20-22]. The ‘available habitat’ quantifies the amount of available habitat (i.e., that can be reached) in the landscape for a particular species, accounting both for the habitat inside an area itself (intra-patch connectivity) and between areas (inter-patch connectivity) [20,21]. We calculated dPC using Conefor Sensinode 2.6 [23], available at <http://www.conefor.org/>. We considered a network among both existing protected areas and proposed conservation areas, and ranked the dPC of the last. Conefor Sensinode requires information about the organisms’ dispersal capacities, which are species-specific, and about distances among areas. We used a 10 km median dispersal distance, appropriate for medium to large dispersers [19], which are the organisms with larger requirements of habitat, and that will potentially benefit the most from higher connectivity [24]. To address distances within elements of the system we calculated ‘effective distances’ among areas using Pathmatrix 1.1 ArcView extension [25]. The human footprint layer and mining concessions were used as resistance surface for distance calculations under the consideration that the movement of the animals is easier across low disturbed areas than through impacted ones and impossible across mines. Thus, the complete set of parameters used in Conefor Sensinode was: nodes file: existing protected areas and proposed conservation areas, connection file: effective distances between nodes calculated with Pathmatrix, PC index with median dispersal distance of 10 km, precision: high.

**REFERENCES**

1. UNEP-WCMC (2004) UNEP-WCMC Species Database. World Conservation Monitoring Center of the United Nations Environment Programme. Available: <http://www.unep-wcmc-apps.org/isdb/Taxonomy/>. Accessed 1 September 2012.

2. Schulenberg TS, Stotz DF, Lane DF, O'Neill JP, Parker TA (2007) Birds of Peru. Princeton: Princeton University Press. 656 p.

3. Pacheco V, Cadenillas R, Salas E, Tello C, Zeballos H (2009) Diversidad y endemismo de los mamiferos del Perú. Revista Peruana de Biología 16: 5-32.

4. IUCN (2011) The IUCN Red List of Threatened Species. Version 2011.1. Available: <http://www.iucnredlist.org/>. Accessed 1 May 2011.

5. Duellman WE, Lehr E (2009) Terrestrial-Breeding Frongs (Strabomantidae) in Peru. Münster: Natur und Tier - Verlag Gmb. 386 p.

6. Rosser N, Phillimore AB, Huertas B, Willmott KR, Mallet J (2012) Testing historical explanations for gradients in species richness in heliconiine butterflies of tropical America. Biological Journal of the Linnean Society 105: 479-497.

7. Munson MA, Webb K, Sheldon D, Fink D, Hochachka WM, et al. (2011) The eBird Reference Dataset, Version 3.0. Database: ebird. Available: <http://ebird.org/content/ebird/>. Accessed 19 April 2011.

8. Brooks TM, Bakarr MI, Boucher T, Da Fonseca GAB, Hilton-Taylor C, et al. (2004) Coverage provided by the global protected-area system: is it enough? BioScience 54: 1081-1091.

9. Elith J, Leathwick J (2009) The contribution of species distribution modelling to conservation prioritization. In: Moilanen A, Wilson KA, Possingham HP, editors. Spatial conservation prioritization Quantitative methods and computational tools. Oxford: Oxford University Press. pp. 70-93.

10. Carvalho SB, Brito JC, Pressey RL, Crespo E, Possingham HP (2010) Simulating the effects of using different types of species distribution data in reserve selection. Biological Conservation 143: 426-438.

11. Phillips SJ, Dudík M, Schapire RE (2004) A maximum entropy approach to species distribution modeling. Proceedings of the 21st International Conference on Machine Learning. New York: ACMPress. pp. 655-662.

12. Hijmans RJ, Cameron SE, Parra JL, Jones PG, Jarvis A (2005) Very high resolution interpolated climate surfaces for global land areas. International Journal of Climatology 25: 1965–1978.

13. Ball IR, Possingham HP, Watts ME (2009) Marxan and relatives: Software for spatial conservation prioritisation. In: Moilanen A, Wilson KA, Possingham HP, editors. Spatial conservation prioritisation: Quantitative methods and computational tools. Oxford, UK: Oxford University Press. pp. 185-195.

14. Game ET, Grantham H (2008) Marxan User Manual: For Marxan version 1.8.10. University if Queensland, St. Lucia, Queensland, Australia and Pacific Marine Analysis and Research Association, Vancouver, British Columbia, Canada. 127 p.

15. Sanderson EW, Jaiteh M, Levy MA, Redford KH, Wannebo AV, et al. (2002) The Human Footprint and the Last of the Wild. Bioscience 52: 891-904.

16. WCS, CIESIN (2005) Last of the Wild Data Version 2, 2005 (LTW-2): Global Human Footprint Dataset (Geographic). Palisades, NY: NASA Socioeconomic Data and Applications Center (SEDAC): Columbia University. Available: http://sedac.ciesin.columbia.edu/data/set/wildareas-v2-human-footprint-geographic. Accessed 30 October 2012.

17. Stewart RR, Possingham HP (2005) Efficiency, costs and trade-offs in marine reserve system design. Environmental Modeling and Assessment 10: 203-213.

18. Brooks TM, Mittermeier RA, Fonseca GABd, Gerlach J, Hoffmann M, et al. (2006) Global biodiversity conservation priorities. Science 313: 58-61.

19. Gurrutxaga M, Rubio L, Saura S (2011) Key connectors in protected forest area networks and the impact of highways: A transnational case study from the Cantabrian Range to the Western Alps (SW Europe). Landscape and Urban Planning 101: 310-320.

20. Saura S, Rubio L (2010) A common currency for the different ways in which patches and links can contribute to habitat availability and connectivity in the landscape. Ecography 33: 523-537.

21. Saura S, Pascual-Hortal L (2007) A new habitat availability index to integrate connectivity in landscape conservation planning: Comparison with existing indices and application to a case study. Landscape and Urban Planning 83: 91-103.

22. Urban D, Keitt T (2001) Landscape connectivity: a graph-theoretic perspective. Ecology 82: 1205-1218.

23. Saura S, Torné J (2009) Conefor Sensinode 2.2: A software package for quantifying the importance of habitat patches for landscape connectivity. Environmental Modelling & Software 24: 135-139.

24. Possingham HP, Wilson KA, Andelman SJ, Vynne CH (2006) Protected Areas. Goals, Limitations, and Design. In: Groom MJ, Meffe GK, Carroll CR, editors. Principles of Conservation Biology. pp. 507 - 549.

25. Ray N (2005) PATHMATRIX: a geographical information system tool to compute effective distances among samples. Molecular Ecology Notes 5: 177-180.
